# The HEART score in predicting major adverse cardiac events in patients presenting to the emergency department with possible acute coronary syndrome: protocol for a systematic review and meta-analysis

**DOI:** 10.1186/s13643-018-0816-4

**Published:** 2018-10-02

**Authors:** Christopher Byrne, Cristian Toarta, Barbra Backus, Tim Holt

**Affiliations:** 1 Department of Medicine, University of Toronto, 190 Elizabeth Street, R. Fraser Elliot Building, Rm 3-805, Toronto, M5G 2C4 Canada; 2 Albert Schweitzer Hospital, Albert Schweitzerplaats 25, 3318 AT Dordrecht, Netherlands; 3 Nuffield Department of Primary Care Health Sciences, Radcliffe Observatory Quarter, University of Oxford, Radcliffe Primary Care Building, Woodstock Road, Oxford, OX2 6GG UK

**Keywords:** Emergency department, Acute coronary syndrome, HEART score, Major adverse cardiac events, Prognosis

## Abstract

**Background:**

Acute coronary syndrome (ACS) is a common, sometimes difficult to diagnose spectrum of diseases occurring after abrupt reduction in blood flow through a coronary artery. Given the diagnostic challenge, it is sensible for emergency physicians to have an approach to prognosticate patients with possible ACS. Multiple prediction models have been developed to help identify patients at increased risk of adverse outcomes. The HEART score is the first model to be derived, validated, and undergo clinical impact studies in emergency department (ED) patients with possible ACS.

**Objective:**

To develop a protocol for a prognostic systematic review of the literature evaluating the HEART score as a predictor of major adverse cardiac events (MACE) in patients presenting to the ED with possible ACS.

**Methods/design:**

This protocol is reported according to the PRISMA-P statement and is registered on PROSPERO. All methodological tools to be used are endorsed by the Cochrane Prognosis Methods Group. Pre-defined eligibility criteria are provided. Multiple strategies will be used to identify potentially relevant studies. Studies will be selected and data extracted using standardised forms based on the CHARMS checklist. The QUIPS tool will be used to assess the risk of bias within individual studies. Outcome measures will include prevalence, risk ratio, and absolute risk reduction for MACE within 6 weeks of ED evaluation, comparing HEART scores 0–3 versus 4–10. HEART score prognostic performance will be evaluated with the concordance (C) statistic (model discrimination), observed to expected events ratio (model calibration), and a decision curve analysis. Reporting biases and methodological, clinical, and statistical heterogeneity will be scrutinised. Unless deemed inappropriate, a meta-analysis and pre-defined subgroup and sensitivity analyses will be performed. Overall judgements about evidence quality and strength of recommendations will be summarised using the GRADE approach.

**Discussion:**

This review will identify, select, and appraise studies evaluating the prognostic performance of the HEART score, producing results of interest to emergency physicians. These results may encourage shared clinical decision-making in the ED by facilitating risk communication with patients and health care providers.

**Systematic review registration:**

PROSPERO 2017 CRD42017084400.

**Electronic supplementary material:**

The online version of this article (10.1186/s13643-018-0816-4) contains supplementary material, which is available to authorized users.

## Background

### Acute coronary syndrome defined

Acute coronary syndrome (ACS) represents a spectrum of diseases occurring after an abrupt reduction in blood flow through a coronary artery and downstream cardiac tissue. Patients with ACS most commonly describe a sudden onset of a pressure-type chest pain occurring at rest or with minimal exertion that may radiate to either or both arms, the neck, or the jaw [1]. Shortness of breath, dizziness, nausea, or sweating can also occur. Clinical presentations differ according to the degree of coronary artery occlusion and subsequent myocardial ischaemia.

Ischaemia involving the full thickness of the heart wall is identified by characteristic electrocardiogram (ECG) findings termed ST elevation. If coronary artery occlusion persists, cardiac tissue is irreparably damaged and markers of cardiac injury become detectable in the blood. When this occurs, the term ST elevation myocardial infarction (STEMI) is used. Myocardial ischaemia and subsequent cardiac injury can also occur in the absence of ST elevation. This characterises non-ST elevation myocardial infarction (NSTEMI). Coronary artery occlusion can also result in a reduction in blood flow not severe enough to produce cardiac injury. This presentation is known as unstable angina (UA) and occurs when symptoms of ACS are present, but markers of cardiac injury are undetectable [1].

### Burden of acute coronary syndrome

Chest pain is the second most common presenting symptom to emergency departments (EDs), accounting for over six million visits in the USA each year [2]. As few as 10% of ED chest pain patients will ultimately be diagnosed with ACS, with many more undergoing prolonged ED observation or hospital admission to rule out ACS [3].

### Emergency department approach

There are no guidelines for what rate of missed ACS is acceptable in emergency medicine practice. Surveys of emergency physicians (EPs) find a large majority desire a miss rate of less than 1% [4]. Acute coronary syndrome can be a challenging diagnosis to confirm or exclude in the ED. There are many alternate explanations for chest pain, an abnormal ECG, or detectable markers of cardiac injury. Conversely, to exclude UA, the clinician must have full confidence in the patient history as, by definition, markers of cardiac injury are undetectable and the ECG may be normal. The term “possible ACS” can be used during initial ED evaluation if elements of the history are of concern, the ECG is unrevealing, and initial cardiac biomarker data are not yet available or undetectable [1]. Given the diagnostic challenge, it is sensible for EPs to have an approach to prognosticate patients with possible ACS. In the absence of a definitive diagnosis, patients perceived to be at unacceptable risk for adverse outcomes can be referred for additional observation and investigation in hospital.

### Risk stratification of possible acute coronary syndrome

Many clinicians naturally incorporate elements from patient’s demographics, risk factors, symptoms, physical exam, and investigations to formulate both diagnostic and prognostic impressions. An alternate approach is to formalise these elements into a prediction model. However, some physicians dismiss prediction models for lacking evidence of superiority when compared to clinical impression [5–7]. Nonetheless, for diagnostic dilemmas such as possible ACS, a formal prognostic prediction model can help EPs decide on management and disposition [8]. Prognostic models may also facilitate communication with patients and other health care providers by synthesising the clinical context and investigations into a quantitative risk assessment [9]. Multiple prediction models have been developed to help identify patients with possible ACS at increased risk of adverse outcomes [8, 10–12]. These models have been applied with variable efficacy and physician uptake in the ED setting.

### The HEART score

The HEART score is one such prediction model. It was designed specifically for short-term risk stratification of patients with possible ACS [8]. Based on clinical experience and interpretation of the medical literature, a group of physicians at a community hospital in the Netherlands expected patient history, ECG abnormalities, higher age, multiple risk factors for coronary artery disease, and elevated cardiac troponin levels to be predictors of major adverse cardiac events (MACE). These represent the five elements of the HEART score (see Table 1). Though deriving a prediction model by expert opinion represents a methodological drawback [13], the five elements and chosen weights of the HEART score are supported by subsequent regression analyses [14].

**Table 1:** Composition of the HEART score for patients in the ED with possible ACS

*H*istory	Highly suspicious	2
	Moderately suspicious	1
	Slightly suspicious	0
*E*CG	Significant ST depression	2
	Non-specific repolarisation disturbance	1
	Normal	0
*A*ge	≥ 65 years	2
	45–65 years	1
	< 45 years	0
*R*isk factors*	≥ 3 risk factors	2
	1 or 2 risk factors	1
	No risk factors	0
*T*roponin	≥ 3× normal limit	2
	1–3× normal limit	1
	≤ normal limit	0
		Total

*Risk factors for coronary artery disease include currently treated diabetes mellitus, current or recent (< 1 month) smoker, diagnosed hypertension, diagnosed hypercholesterolaemia, family history of coronary artery disease, and obesity

### Why the HEART score is important

There are alternate prognostic prediction models in patients with possible ACS to undergo derivation and validation in ED patients [11, 12]. However, the HEART score is the only model to be evaluated by multiple independent research groups in both validation and clinical impact studies [15–20]. In addition, the HEART score outperforms alternate prediction models in comparison studies [15, 21]. The HEART score is also intuitive to the EP, relying on elements of clinical experience rather than the sometimes less accessible, yet statistically valid predictors seen in other models [8].

### The current literature and its limitations

A systematic review and meta-analysis involving the HEART score was published in May 2017 [22]. The objective of this review was to summarise the evidence on the diagnostic accuracy of the HEART score for predicting MACE in patients presenting to the ED with possible ACS. The target condition of MACE (see Table 2) was defined as a composite of myocardial infarction (MI), percutaneous coronary intervention (PCI), coronary artery bypass graft (CABG), and all-cause death.

**Table 2:** Definitions of major adverse cardiac events [42]

Myocardial infarction	Colloquially known as a “heart attack”, occurs when blood flow decreases or stops to a part of the heart, causing irreversible damage to the heart muscle.
Percutaneous coronary intervention	Non-surgical procedure used to treat narrowed coronary arteries of the heart found in coronary artery disease. Most commonly, a balloon catheter is inserted into a diseased coronary artery and inflated to relieve the narrowing. A stent can then be deployed to keep the vessel open.
Coronary artery bypass graft	Surgical procedure used to treat narrowed coronary arteries of the heart found in coronary artery disease. A redundant blood vessel is harvested from another part of the body and attached in such a way that a diseased coronary artery is bypassed.

Cochrane methodology for diagnostic test accuracy systematic reviews were applied. Randomised controlled trials and both retrospective and prospective observational studies were eligible for inclusion. To be included, studies were required to evaluate the HEART score upon ED arrival and report the number of MACE over the study period. The literature search identified 18 studies as potentially eligible for inclusion. Following an independent review, 11 studies met the inclusion criteria and provided data permitting the calculation of diagnostic accuracy measures. Two studies were excluded from meta-analysis owing to concerns surrounding deviation from how the original HEART score was defined. A total of 9 studies and 11,217 patients were included in the primary meta-analysis.

The authors found the overall pooled prevalence of MACE to be 15.4% (95% confidence interval (CI) 14.8–16.1%, range 7.3–29.1%) at a mean follow-up time of 6 weeks. Among 4101 patients categorised as low risk and suitable for early ED discharge (HEART score 0–3), the pooled prevalence of MACE was 1.6% (95% CI 1.2–2.0%). Results were otherwise presented using measures of diagnostic accuracy, including sensitivity, specificity, and predictive values. The pooled sensitivity and specificity of the HEART score for predicting MACE were 96.7% (95% CI 94.0–98.2%) and 47.0% (95% CI 41.0–53.5%), respectively.

#### A problematic reference standard

In any diagnostic accuracy study, the index test (test under evaluation) and reference standard (best available method for establishing presence or absence of target condition) should be described in sufficient detail to permit replication [23]. In this review, HEART score 0–3 represented a negative index test and HEART score 4–10 represented a positive index test in calculating measures of diagnostic accuracy (see Table 3). This cut-off was selected as the authors considered patients with HEART score 0–3 at low risk of developing MACE and potentially eligible for immediate discharge from the ED.

**Table 3:** The HEART score as an index test for identifying MACE, presented in two by two table

		Target condition MACE	
		Present	Absent
Index test HEART score	Positive HEART 4–10	True positive	False positive
	Negative HEART 0–3	False negative	True negative

The authors defined the reference standard as follows:

The third universal definition of AMI, consistent with a rise and/or fall of a cardiac biomarker with minimally one result above the 99th percentile upper reference limit in the context of a patient presenting with cardiac ischaemia.

**Fig. 1:**
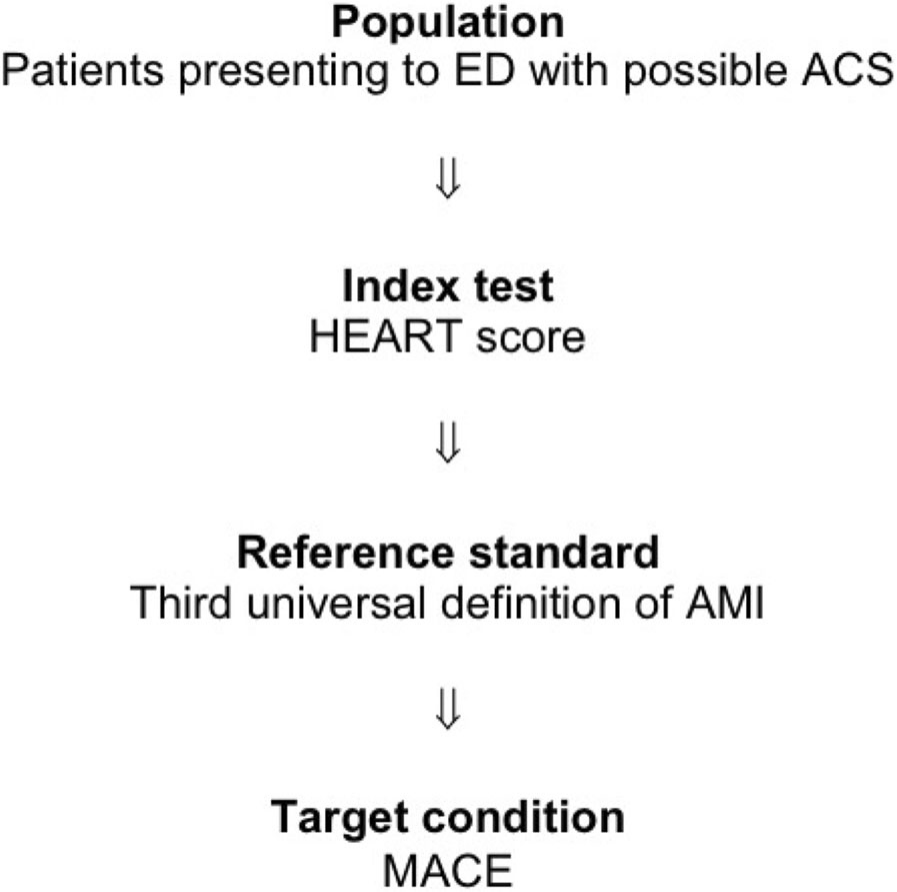
Diagnostic accuracy study design eligible for inclusion as described in the “Methods/design” section of the systematic review

The index test and reference standard descriptions illustrate the basic diagnostic accuracy study design eligible for inclusion in this review (see Fig. 1).

The rationale for choosing this reference standard was not provided. If one chooses to conceptualise the HEART score as a diagnostic test, the passage of time seemingly represents a more reliable standard for detecting MACE. The authors suggest that to diagnose or predict future MACE, the reference standard is the “third universal definition of AMI”. This is problematic because the diagnosis of MI by a rise and/or fall of troponin is also, by definition, a MACE. This diagnostic accuracy systematic review’s reference standard is thus also part of its target condition.

#### Incorporation and verification biases

**Fig. 2:**
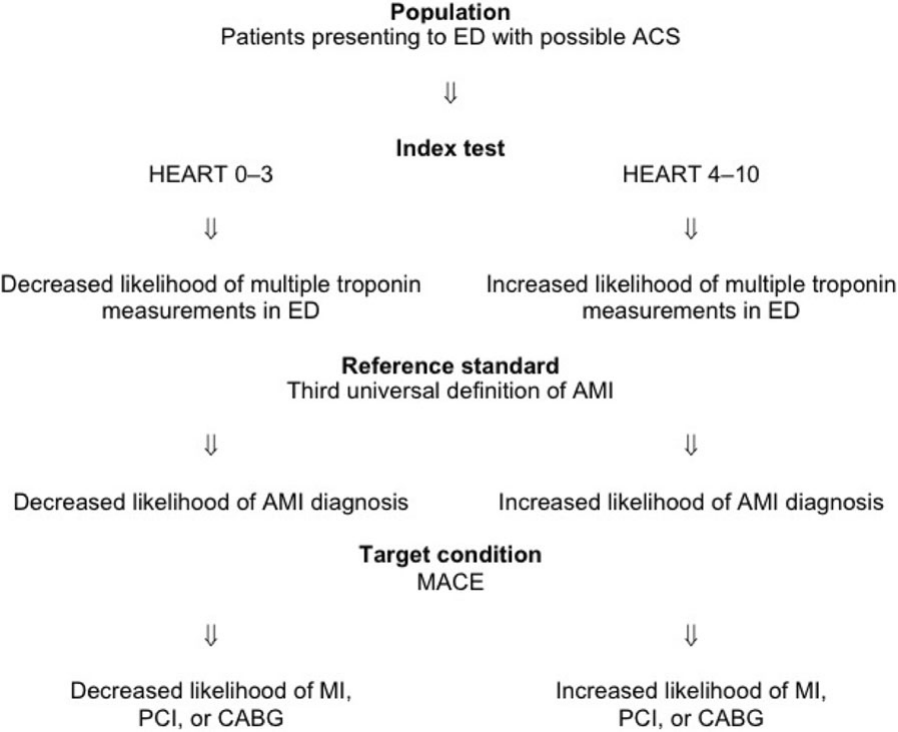
Illustration of verification bias in diagnostic accuracy studies of the HEART score

The reference standard also introduces incorporation and verification biases [24]. Troponin levels are components of both the HEART score and reference standard. This incorporation bias elevates the risk of overstating both the sensitivity and specificity of the HEART score. Experience and clinical practice guidelines suggest patients with a history concerning for ACS, an abnormal ECG, advanced age, or many risk factors for coronary artery disease are very likely to have multiple troponin levels measured while in the ED [1]. These patients will also have a high HEART score. When multiple troponin levels are measured over a period of ED observation, a patient is more likely to have a positive result, be diagnosed with AMI, and subsequently undergo revascularisation by PCI or CABG (see Fig. 2). This verification bias similarly risks overstating both the sensitivity and specificity of the HEART score. A false-negative HEART score 0–3 is less likely to occur if only one troponin measurement occurs. Likewise, a false-positive HEART score 4–10 is less likely when multiple troponin measurements occur. The impact of these biases could have been explored via a sensitivity analysis comparing trials standardising an observation period or multiple troponin levels for all participants irrespective of HEART score (lower risk of bias) to those trials entrusting that decision to the discretion of the treating physician (higher risk of bias).

#### Importance of outcome blinding in retrospective studies

Several large studies have been published beyond the search window of the May 2017 review, including at least one randomised trial and multiple prospective observational studies [17, 25–27]. Nine of the 11 studies included in this review were retrospective in nature. In general, retrospective studies of the HEART score have a higher risk of bias [28]. For example, lack of outcome blinding combined with unclear predictor measurement criteria might encourage an assessor to rate the history as “highly suspicious” if a patient was known to have sustained a MACE. This bias likely overestimates the predictive value of the HEART score and should be explicitly addressed in any systematic review. The importance of this bias could have been evaluated by comparing trials with clearly stated outcome blinding (lower risk of bias) to those lacking outcome blinding (higher risk of bias) via a sensitivity analysis. Alternatively, the authors could have made trials without outcome blinding ineligible for inclusion in the review.

#### Maximising external validity for emergency physicians

The external validity of many observational studies of the HEART score is also of concern. For example, it is debatable whether history and ECG scoring by a researcher or cardiologist reviewing patient charts is reliable as a proxy of how the history and ECG would have been scored by the attending EP. In the context of this review, the strongest study design to evaluate the utility of the HEART score as a predictor of MACE would be a prospective observational study mandating the attending clinician at the time of initial ED assessment determine the HEART score. Studies incorporating this design should be emphasised in a prognostic systematic review. Similarly, across studies included in the May 2017 systematic review, there are potentially important differences in the baseline risk of MACE (range 7.3–29.1%) and type of troponin assays used [22]. The reviewers could have better assessed the impact of clinical heterogeneity in study populations and HEART score measurement by subgroup analyses.

### Rationale for this review

In summary, the HEART score is a user-friendly prediction model for clinicians assessing patients presenting to the ED with possible ACS. While some have opted to evaluate the HEART score as a diagnostic accuracy test [22], this perspective is challenged by an uncertain diagnostic reference standard. In addition, the impact of incorporation, verification, and outcome blinding biases with the potential to overstate the score’s predictive performance has not been fully explored. The HEART score was originally designed to be a prognostic prediction model, utilising information from the patient history, ECG, age, risk factors, and troponin measurement at the initial ED assessment [8]. A systematic review incorporating methodology specifically for prognostic prediction models is thus warranted. This review should aim to evaluate the impact of biases in HEART score and outcome measurement and produce results generalisable to EPs applying variable clinical approaches (e.g. type of troponin assay used) across ED populations with variable baseline risks of MACE.

### Objective

The objective is to develop a protocol for a systematic review and meta-analysis of the literature evaluating the HEART score as a prognostic predictor of MACE in patients presenting to the ED with possible ACS.

## Methods/design

This systematic review protocol is reported according to the Preferred Reporting Items for Systematic Review and Meta-Analysis Protocols (PRISMA-P) statement [29]. The review protocol was registered on the PROSPERO website (http://www.crd.york.ac.uk/PROSPERO/display_record.php?ID=CRD42017084400) on December 19, 2017 (CRD42017084400). All methodological tools to be used in this review are endorsed by the Cochrane Prognosis Methods Group.

### Criteria for considering studies for this review

The inclusion and exclusion criteria for this review are summarised below.

**Table:** 

Eligibility criteria for the systematic review	
Inclusion criteria	Exclusion criteria
Studies	
Original research Retrospective observational study, prospective observational study, or randomised trial	Derivation or internal validation study Retrospective observational study with lack of or uncertain outcome blinding
Participants	
Patients presenting to ED or chest pain unit Symptoms of ACS^a^ present or assessing clinician considering ACS as diagnosis Diagnostic workup includes ECG and troponin measurement	Study evaluates or reports xon only patients with HEART score 0–3 or a subset of the population of interest Study excludes patients who sustain a MACE while in the ED or chest pain unit^b^
Intervention	
HEART score determined from data obtained at initial physician assessment	
Primary outcome	
MACE, a composite outcome including death, MI, PCI, or CABG Primary outcome can be stratified by HEART score 0–3 and 4–10 Outcome occurs within 6 weeks of ED or chest pain unit assessment	

^a^Symptoms of ACS include chest, arm, or jaw pain; shortness of breath; dizziness; nausea; or sweating

^b^An exception will be made if a study excludes patients with definite STEMI or ACS at initial assessment as diagnostic uncertainty is lacking, and these patients are typically immediately transferred to the nearest cardiac catheterisation facility

#### Types of studies

Original research articles on the external validation of the HEART score will be eligible for this review. Derivation and interval validation studies will be excluded. Randomised controlled trials, prospective observational studies, and retrospective observational studies will be considered for inclusion. For a retrospective study to be included, assessors of the patient history and ECG must be blinded to the outcome of MACE. If this is not clearly stated in the study’s methodology, authors will be contacted to clarify if assessors were suitably blinded. If uncertainty regarding blinding persists following an attempt to contact study authors, the study will be excluded from this review. There will be no restrictions on language or timeframe of publication.

#### Types of participants

Studies evaluating patients with possible ACS (see Table 4) at initial physician assessment in an ED or chest pain unit will be considered for inclusion. Chest pain units exist in some, but not all jurisdictions. These units are typically located in or near the ED and provide a setting for observation and investigation of acute chest pain patients [30]. As our population of interest includes all patients presenting to an ED with possible ACS, studies evaluating only a subset of the population of interest (e.g. only patients with HEART score 0–3) will be excluded. Similarly, studies failing to include all patients who sustain a MACE while in the ED or chest pain unit will also be excluded. An exception will be made if a study excludes patients with definite STEMI or ACS at initial assessment as diagnostic uncertainty is lacking, and these patients are typically immediately transferred to the nearest cardiac catheterisation facility.

**Table 4:** Definition of possible ACS

Possible ACS	Symptoms of ACS present (chest, arm, or jaw pain; shortness of breath; dizziness; nausea; or sweating) or assessing clinician considering ACS as a possible diagnosis, and initial diagnostic workup includes both an ECG and troponin measurement.

#### Types of interventions

All patients must have a HEART score determined using data obtained at the initial physician assessment.

#### Types of comparisons

The types of comparisons are not applicable.

### Types of outcome measures

#### Primary outcomes

The primary outcome will be MACE within 6 weeks of initial ED or chest pain unit assessment. Major adverse cardiac events are a composite outcome encompassing death, MI, PCI, or CABG. The primary outcome will be stratified by HEART score 0–3 and HEART score 4–10.

The intent of the HEART score is to identify a low-risk group of patients that can be safely discharged from the ED. HEART score 0–3 represents the low-risk group as defined in the prediction model’s derivation study. The authors of the May 2017 review agreed with this stratification approach, thus identifying 4101 of 11,217 (36.6%) patients as low risk in their analysis [22]. The pooled prevalence of MACE in these 4101 patients was 1.6% (95% CI 1.2–2.0%). This seems a sensible risk level at which to initiate a shared clinician-patient decision on discharge versus continued hospital observation or investigation. While alternate stratification strategies may further lower the risk of MACE, this must be balanced by subjecting a higher proportion of patients to unnecessary observation and investigation.

#### Secondary outcomes

Secondary outcomes will be death within 6 weeks of initial ED or chest pain unit assessment and MI within 6 weeks of initial ED or chest pain unit assessment. If a study does not include sufficient information to determine secondary outcomes, the authors will be contacted. If after attempting to contact study authors this information remains unavailable, the study will be excluded from secondary outcome analysis. Secondary outcomes will also be stratified by HEART score 0–3 and HEART score 4–10.

### Search methods for identification of studies

Multiple strategies will be used to identify potentially relevant studies, including electronic searches, hand searches of reference lists and conference proceedings, and contacting content experts. A preliminary search was conducted on November 28, 2017. The final search will be conducted in July 2018.

#### Electronic searches

The electronic database search will include the following:

- MEDLINE using PubMed;
- EMBASE using OvidSP;
- Cumulative Index of Nursing and Allied Health Literature (CINAHL);
- Web of Science (WoS) (all databases);
- Cochrane Central Register of Controlled Trials (CENTRAL);
- Cochrane Database of Systematic Reviews (CDSR);
- NHS Database of Abstracts of Reviews of Effects (DARE); and
- NIHR Health Technology Assessment (HTA) Programme.

ClinicalTrials.gov, the ISRCTN registry, the World Health Organization International Clinical Trials Registry Platform (ICTRP), and PROSPERO will be searched for unpublished and ongoing trials. Databases will be searched using the free text terms “HEART score” or “HEART pathway” without field, language, or date of publication limitations. The search strategy with its corresponding preliminary results is provided in Additional file 1: Appendix S1.

#### Additional search strategies

Reference lists for primary studies eligible for inclusion and review articles will be scrutinised to identify potentially relevant citations. Conference proceedings from the Canadian Association of Emergency Physicians (CAEP), American College of Emergency Physicians (ACEP), Society for Academic Emergency Medicine (SAEM), and International Conference on Emergency Medicine (ICEM) will be hand-searched. The conference proceedings search will be restricted to start in 2008, the year the HEART score was first derived and published

### Data collection and analysis

#### Selection of studies

Results from the search strategies will be combined into a reference manager programme with duplicates excluded. Two authors (CB, CT) will perform the title and abstract screening, excluding obviously ineligible studies. From the potentially relevant articles identified, two authors (CB, CT) will independently perform the full-text reviews and select trials for inclusion using a standardised article inclusion form (see Additional file 1: Appendix S2). Disagreements will be resolved by discussion to reach consensus or third-party adjudication (TH). A list of included trials will be completed (see Additional file 1: Appendix S3).

#### Data extraction and management

Two authors (CB, CT) will independently extract the data from the included studies using an electronic data extraction form (see Additional file 1: Appendix S4). Disagreement not resolved by consensus will be adjudicated by a third author (TH). The data extraction form is based on the Checklist for Critical Appraisal and Data Extraction for Systematic Reviews of Prediction Modelling Studies (CHARMS) [13], modified according to the scope of this review (i.e. external validation studies). This checklist provides a framework for extracting key information from studies of prediction models.

#### Assessment of risk of bias in included studies

To assess the risk of bias within individual studies, the Quality in Prognosis Studies (QUIPS) tool for prognostic studies will be used [28]. Five bias domains (see Table 5) will be rated as having a low, moderate, or high risk of bias according to QUIPS tool criteria. Two independent reviewers (CB, CT) will assess every included study for bias. Disagreements will be resolved by discussion or third-party adjudication (TH). Results from the risk of bias assessment will be presented in table format with colour coding for easy visualisation.

**Table 5:** Bias domains to be assessed using the QUIPS tool

Domain	Optimal study
Study participation	Study sample adequately represents the population of interest That is, relationship between HEART score and MACE unlikely to be different for participants and eligible non-participants
Study attrition	Study data available (i.e. participants not lost to follow-up) adequately represent study sample That is, relationship between HEART score and MACE unlikely to be different for completing and non-completing participants
Prognostic factor measurement	Prognostic factors measured in a similar way for all participants That is, measurement of HEART score unlikely to be different if MACE is present versus absent
Outcome measurement	Outcomes of interest measured in a similar way for all participants That is, measurement of MACE unlikely to be different with varying HEART scores
Statistical analysis and reporting	Statistical analysis appropriate, and all primary outcomes reported That is, reported results unlikely to be spurious or biased due to analysis or reporting

#### Data analysis and measures of prediction model performance

Descriptive statistics will be presented as means with standard deviations (SD) and medians with interquartile ranges (IQR). Outcomes in this review are dichotomous and will be presented as pooled prevalences, Mantel-Haenszel risk ratios (RRs), and absolute risk reductions (ARRs) with 95% confidence intervals (CI). Prediction model performance will be summarised using measures of discrimination, calibration, and a decision curve analysis (DCA).

Discrimination refers to a prediction model’s ability to distinguish between patients developing and not developing the outcome [31]. In the context of this review’s primary outcome, the C-statistic provides the probability a randomly selected patient who experienced a MACE had a higher risk HEART score than a patient who had not experienced a MACE. The C-statistic is equal to the area under the receiver operating characteristic (ROC) curve.

Calibration refers to a model’s accuracy of predicted risk probabilities, indicating the extent to which expected and observed outcomes agree [31]. Summarising estimates of calibration is difficult because calibration plots are often not presented, and studies tend to report different types of summary statistics in calibration [32]. As a result, calibration will be quantified by individual study and summary observed to expected events ratios (O:E events ratios) with 95% confidence intervals stratified by HEART scores of 0–3 and 4–10. The summary O:E events ratio provides a rough indication of overall model calibration across the entire range of predicted risks [31].

Decision curve analysis (DCA) is a method for evaluating a prognostic tool with competing benefits and harms across a range of patient preferences and risk tolerances [33]. The decision to observe or continue to investigate a patient with possible ACS depends on how confident the clinician is in a patient’s prognosis, the efficacy and complications of additional observation or investigation, and the patient’s willingness to accept the burden of an observation or investigation plan that might be unnecessary. To address these clinical decision challenges, DCA uses a measure called the threshold probability [34].

In this review, the threshold probability represents the probability of MACE at which an individual believes the harms of unnecessary observation or investigation (e.g. coronary angiogram) if MACE will not occur are equal to the benefits of observation or investigation if MACE will occur (e.g. early recognition and treatment of MI or obstructive coronary artery disease by PCI or CABG). The probability threshold will vary from individual to individual. If additional observation or investigation is perceived by a patient to have high value and minimal morbidity, inconvenience, and cost, the threshold probability for proceeding with this management plan will be low. Conversely, if this plan is perceived to be minimally effective or associated with adverse effects, the threshold probability will be high [35]. Threshold probabilities thus provide a framework for processing benefits and harms into a patient-centred, evidence-based clinical decision.

**Fig. 3:**
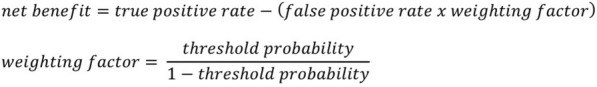
Net benefit calculation for decision curve analysis

This review’s DCA will compare the following approaches: (1) observe or investigate all patients presenting to ED with possible ACS regardless of HEART score or (2) observe or investigate only those patients with HEART score 4–10. In this DCA, the desirable outcome or “benefit” is additional observation or investigation for those patients who will have a MACE (true positives). The undesirable outcome or “harm” is additional observation or investigation for those patients who will not have a MACE (false positives). The net benefit was calculated by determining the difference between the expected benefit and the expected harm. The expected benefit is represented by the proportion of patients who will have a MACE and be observed or investigated (true-positive rate). The expected harm is represented by multiplying the false-positive rate by a weighting factor based on the patient’s threshold probability (see Fig. 3) [35].

For example, if a patient’s threshold probability of a MACE is 10%, the weighting factor applied to the proportion of patients observed or investigated who will not have a MACE would be 0.1/0.9, or one ninth. This minimises the effect of false-positive results because the burden of an unnecessary management plan is perceived by the patient to be low. Graphically, the DCA is expressed as a curve, with a net benefit score on the vertical axis and risk thresholds on the horizontal axis. This analysis is intended to help tailor clinical decision-making to an individual patient’s threshold probability.

#### Assessment of heterogeneity

Heterogeneity will be assessed by analysing methodological, clinical, and statistical diversity.

Methodological diversity will be judged primarily by the risk of bias assessment as per the QUIPS tool (see Table 5). Sensitivity analyses will be performed to assess the robustness of results after accounting for the impact of subjective methodological assumptions and inclusion of studies at high risk of bias (see the “Sensitivity analysis” section).

Anticipated sources of important clinical diversity include variable baseline risks of MACE, use of conventional/contemporary versus high-sensitivity troponin assays, and attending clinician versus researcher or non-attending clinician determined HEART scores. Subgroup analyses will be performed to explore the impact of clinical heterogeneity (see the “Subgroup analyses” section).

Statistical heterogeneity will be visually displayed in forest plots, with a poor overlap of confidence intervals for the results of individual studies indicating the presence of heterogeneity [36]. More formally, statistical heterogeneity will be assessed using the chi-squared (*χ*^2^) test and *I*^2^ statistic. The *χ*^2^ test assesses whether observed differences in the results are compatible with chance alone [36]. A low *P* value (or large *χ*^2^ statistic) provides evidence of variation in effect estimates beyond chance [36]. The *I*^2^ statistic quantifies the percentage of total variability in effect estimates that is due to heterogeneity rather than chance [36]. A rough guide to interpretation of the *I*^2^ statistic is as follows: 0 to 40% might not be important, 30 to 60% may represent moderate heterogeneity, 50 to 90% may represent substantial heterogeneity, and 75 to 100% represents considerable heterogeneity [36]. There are challenges in interpreting the *I*^2^ statistic in the context of prognostic studies, where large sample sizes of included studies result in very narrow confidence intervals. As a result, *I*^2^ for pooled risk estimates can be extremely high even in the presence of modest inconsistency in risk estimates between individual studies [37]. If statistically significant and considerable heterogeneity exists (*P* < 0.10 and *I*^2^ > 75%) and a meta-analysis is nonetheless deemed appropriate, rationale for performing a meta-analysis with potential explanations for statistical heterogeneity (e.g. differences in the prevalence of MACE between studies) will be explored.

#### Assessment of reporting biases

Reporting biases arise when the dissemination of research findings is influenced by the nature and direction of results [36]. A funnel plot will be generated if at least 10 studies are included in the meta-analysis, with measures of effect size plotted on the horizontal axis and the standard errors of these measures plotted on the vertical axis. To evaluate reporting biases, funnel plot symmetry will be visually inspected, with measures of effect size plotted on the horizontal axis and the standard errors of these measures plotted on the vertical axis. Evidence of small study effects will be assessed with Egger’s test and visually displayed as an Egger’s plot.

#### Data synthesis

Meta-analyses implementing a random effects model will be performed. External validation studies typically differ in design, execution, and case-mix [38]. Random effects models allow for the presence of heterogeneity between studies by assuming that the effects being estimated in different studies are not identical, but follow some distribution [36]. Mantel-Haenszel RR and ARR analyses will be conducted using Review Manager (RevMan) software [39]. The summary ROC curve and C-statistic, pooled O:E events ratio, and DCA analyses will be conducted using Stata/IC software applying MIDAS and DCA commands [40].

#### Subgroup analyses

As the prognostic performance of the HEART score in patients presenting to the ED with possible ACS may depend on the baseline risk of MACE, the troponin assay utilised, and who determines the HEART score, the following subgroup analyses will be performed: (1) low versus intermediate versus high baseline risk of MACE, (2) conventional/contemporary versus high-sensitivity troponin assay (see Table 6) used, and (3) attending clinician determined versus researcher or non-attending clinician determined HEART score. These subgroups were selected to maximise the generalisability of results to variable ED clinical approaches and populations.

**Table 6:** Definition of high-sensitivity cardiac troponin assay [43]

High-sensitivity troponin assay	Total imprecision (as per the coefficient of variation) < 10% at the 99th percentile value of a healthy reference population and limit of detection permitting measurable concentrations for at least 50% of healthy individuals.

#### Sensitivity analysis

A sensitivity analysis is a repeat of a meta-analysis, substituting alternate decisions or ranges of values that are subjective in nature [36]. For example, some investigators have deviated from the definition of a low-risk HEART score as described in the score’s derivation study (HEART score 0–3), instead of defining low risk as those with HEART score 0–2 [22]. There have also been variable approaches to outcome measurement, with some studies standardising an observation period or multiple troponin measurements regardless of HEART score (lower risk of bias) and other studies leaving that decision to the discretion of the treating physician (higher risk of bias). Similarly, some HEART score studies assess the primary outcome of MACE at 30 days, while others assess for this outcome at 6 weeks [22]. To test the robustness of this review’s findings, the following sensitivity analyses will be performed: (1) low versus moderate versus high risk of bias assessment, (2) low-risk HEART score of 0–2 versus 0–3, and (3) primary outcome of MACE assessed at 30 days versus 6 weeks.

## Summary of findings

The Grading of Recommendations, Assessment, Development and Evaluation (GRADE) approach to making judgements about the quality of evidence and strength of recommendations was initially developed for therapeutic questions but can be applied to bodies of evidence estimating prognosis [37, 41]. This system will be used to assess the quality of evidence in this review. As per the GRADE approach, the following assessment criteria will be used when upgrading or downgrading confidence in the results of this review: (1) risk of bias, (2) inconsistency in results, (3) imprecision of results, (4) indirectness (i.e. generalisability or applicability of results), and (5) publication bias (see Table 7). The assessments will be performed by two independent reviewers (CB, CT) independently. Disagreements will be resolved by discussion or third-party adjudication (TH).

**Table 7:** Definitions of levels of evidence about prognosis [37]

Quality level	Optimal study
High	We are very confident that the true prognosis lies close to that of the estimate.
Moderate	We are moderately confident that the true prognosis is likely to be close to the estimate, but there is a possibility that it is substantially different.
Low	Our confidence in the estimate is limited; the true prognosis may be substantially different from the estimate.
Very low	We have very little confidence in the estimate; the true prognosis is likely to be substantially different from the estimate.

## Discussion

This review will identify, select, and appraise studies evaluating the prognostic performance of the HEART score, producing results of interest to physicians caring for patients with possible ACS in an ED or similar setting. It is our hope this review will increase the precision of existing HEART score literature through meta-analyses of included studies. Exploration of pre-specified subgroup effects may improve confidence in the applicability of the HEART score across varied clinical settings. In addition, this review will contribute to the knowledge translation process and may inform future clinical practice guidelines. The results of this review may also identify research gaps or generate new hypotheses related to the evaluation and management of patients with possible ACS. Most importantly, we hope this review will encourage a model of shared clinical decision-making in the ED by facilitating risk communication with patients and between health care providers.

## Additional file

Additional file 1: **Appendix S1.** Search strategy. **Appendix S2.** Article inclusion form. **Appendix S3.** Studies included in review. **Appendix S4.** Data extraction form. (DOCX 43 kb)

## Abbreviations

ACS: Acute coronary syndrome
AMI: Acute myocardial infarction
CABG: Coronary artery bypass graft
CHARMS: Checklist for Critical Appraisal and Data Extraction for Systematic Reviews of Prediction Modelling Studies
ED: Emergency department
EP: Emergency physician
GRADE: Grading of Recommendations, Assessment, Development and Evaluation
MACE: Major adverse cardiac event
MI: Myocardial infarction
PCI: Percutaneous coronary intervention
QUIPS: Quality in prognosis studies
