# Supplementary material for: The HEART score in predicting major adverse cardiac events in patients presenting to the emergency department with possible acute coronary syndrome: protocol for a systematic review and meta-analysis
Source: Syst Rev. 2018 Oct 2;7:148. doi: 10.1186/s13643-018-0816-4 (PMC6169026; doi:10.1186/s13643-018-0816-4)
Supplement: Supplementary file 1 — Appendix S1. Search strategy. Appendix S2. Article inclusion form. Appendix S3. Studies included in review. Appendix S4. Data extraction form. (DOCX 43 kb) [file 13643_2018_816_MOESM1_ESM.docx]

# **Appendix S1: Search Strategy**

**All electronic databases**

Free text search of all fields, with no date or language restrictions: “HEART score” OR “HEART pathway”.

**Preliminary electronic database search (performed on November 28, 2017)**

- MEDLINE using PubMed: 192 results
- EMBASE using OvidSP: 370 results
- Cumulative Index of Nursing and Allied Health Literature (CINAHL): 47 results
- Web of Science (WoS) (All Databases): 204 results
- Cochrane Central Register of Controlled Trials (CENTRAL): 28 results
- Cochrane Database of Systematic Reviews (CDSR): 4 results
- NHS Database of Abstracts of Reviews of Effects (DARE): 0 results
- NHS Health Technology Assessment (HTA) Programme: 0 results
- ClinicalTrials.gov: 18 results
- ISRCTN Registry: 2 results
- World Health Organization International Clinical Trials Registry Platform (ICTRP): 13 results
- PROSPERO: 0 results

# **Appendix S2: Article Inclusion Form**

| **The HEART score in predicting major adverse cardiac events in patients presenting to the emergency department with possible acute coronary syndrome: Criteria for inclusion in systematic review** | | |
| --- | --- | --- |
| **Citation Number**: ______ **Reviewer: CB CT** | | |
| Please assess the following for each paper. **ALL** criteria must be met for study to be eligible for inclusion. Authors should be contacted if additional information is needed to reach final decision. | | |
| **PART 1: Design** | | |
| YES | NO or UNCLEAR | Original research |
| YES | NO or UNCLEAR | Retrospective observational study, prospective observational study, or randomised trial |
| **EXCLUDE** if:  [ ] Derivation or internal validation study  [ ] Retrospective observational study with lack of or uncertain outcome blinding | | |
| **PART 2: Population** | | |
| YES | NO or UNCLEAR | Patients presenting to ED or chest pain unit |
| YES | NO or UNCLEAR | Symptoms of ACS present or assessing clinician considering ACS as diagnosis |
| YES | NO or UNCLEAR | Diagnostic work-up includes ECG and troponin measurement |
| **EXCLUDE** if:  [ ] Study evaluates only patients with HEART score 0–3  [ ] Study excludes patients who sustain a MACE while in the ED or chest pain unit   Exception: definite STEMI/ACS at initial assessment | | |
| **PART 3: Intervention** | | |
| YES | NO or UNCLEAR | HEART score determined from data obtained at initial physician assessment |
| **PART 4: Primary Outcome** | | |
| YES | NO or UNCLEAR | MACE, a composite outcome including death, MI, PCI, or CABG |
| YES | NO or UNCLEAR | Primary outcome can be stratified by HEART score 0–3 and 4–10 |
| YES | NO or UNCLEAR | Outcome occurs within six weeks of ED or chest pain unit assessment |
| **PART 5: Final Decision** | | |
| [ ] Include [ ] Exclude | | |

#

# **Appendix S3: Studies Included in Review**

| **The HEART score in predicting major adverse cardiac events in patients presenting to the emergency department with possible acute coronary syndrome: List of studies included in systematic review** | | | | | | |
| --- | --- | --- | --- | --- | --- | --- |
| **Report**  **ID** | **Lead Author**  **Year** | **Source** | **Country** | **Study**  **Design** | **Number of**  **Sites** | **Study**  **Period** |
| 1 | Poldervaart  2017 | Annals of  Internal Medicine | Netherlands | Randomised  Trial | 9 | 1 Jul 2013 –  31 Aug 2014 |
| .  .  . |  |  |  |  |  |  |

# **Appendix S4: Data Extraction Form**

| **The HEART score in predicting major adverse cardiac events in patients presenting to the emergency department with possible acute coronary syndrome: Data extraction form** | | |
| --- | --- | --- |
| **General Information** | | |
| **Reviewer CB CT** | | |
| **Date form completed**  *(dd/mm/yyyy)* | | |
| **Report ID**  *(as per “studies included in review” form)* | | |
| **Report title**  *(title of paper/abstract/report data extracted from)* | | |
| **Lead author and year report completed** | | |
| **Author contact information** | | |
| **Report type**  *(e.g., journal article, conference abstract)* | | |
| **Report source**  *(e.g., name of journal, name of conference)* | | |
| **Funding sources**  *(including role of funders)* | | |
| **Possible conflicts of interest**  *(for study authors)* | | |
| **Notes:** | | |
| **Source of data** | **Description** *(as in report)* | **Location** *(page/figure/table)* |
| **Source of data**  *(e.g., retrospective cohort, prospective cohort, randomised trial)* |  |  |
| **Participants** | **Description** *(as in report)* | **Location** *(page/figure/table)* |
| **Inclusion criteria** |  |  |
| **Exclusion criteria** |  |  |
| **Recruitment method**  *(e.g., consecutive participants)* |  |  |
| **Location**  *(e.g., Canada)* |  |  |
| **Number of centres** |  |  |
| **Setting**  *(e.g. 265-bed community hospital)* |  |  |
| **Notes:** |  |  |
| **Outcomes to be predicted** | **Description** *(as in report)* | **Location** *(page/figure/table)* |
| **Definition of outcome**  *(e.g., major adverse cardiac event, a composite outcome including…)* |  |  |
| **Was the same outcome definition used in all participants?**  *(yes/no/unclear)* |  |  |
| **Method of outcome measurement** |  |  |
| **Was the same method of outcome measurement used in all participants?** *(yes/no/unclear)* |  |  |
| **Type of outcome**  *(e.g., single or combined endpoints)* |  |  |
| **Was the outcome assessed without knowledge of the candidate predictors (i.e., blinded)?**  *(yes/no/unclear)* |  |  |
| **Were candidate predictors part of the outcome?**  *(yes/no/unclear)* |  |  |
| **Duration of follow-up**  *(e.g., 30 days)* |  |  |
| **Notes:** |  |  |
| **Candidate predictors** | **Description** *(as in report)* | **Location** *(page/figure/table)* |
| **Definition of *History***   - *0 points* - *1 point* - *2 points*   **Method of measurement** |  |  |
| **Definition of *Electrocardiogram***   - *0 points* - *1 point* - *2 points*   **Method of measurement** |  |  |
| **Definition of *Age***   - *0 points* - *1 point* - *2 points*   **Method of measurement** |  |  |
| **Definition of *Risk factors***   - *0 points* - *1 point* - *2 points*   **Method of measurement** |  |  |
| **Definition of *Troponin***   - *0 points* - *1 point* - *2 points*   **Method of measurement** |  |  |
| **Was the HEART score calculated by the attending clinician?**  *(yes/no/unclear)* |  |  |
| **Was the HEART score calculated retrospectively or prospectively?**  *(retrospective/prospective/*  *unclear)* |  |  |
| **Was a high-sensitivity troponin assay used?**  *(yes/no/unclear)* |  |  |
| **Troponin assay utilised with cut-off value** |  |  |
| **Serial troponin data available**  *(yes/no/unclear)* |  |  |
| **Timing of predictor measurement**  *(e.g., upon presenting to ED)* |  |  |
| **Were predictors assessed blinded for outcome?**  *(yes/no/unclear)* |  |  |
| **Were predictors assessed blinded for each other?**  *(yes/no/unclear)* |  |  |
| **Handling of predictors in the modelling**  *(e.g., continuous, linear, categorised)* |  |  |
| **Notes**: | | |
| **Sample size** | **Description** *(as in report)* | **Location** *(page/figure/table)* |
| **Number of participants** |  |  |
| **Number of outcomes**   - *Major adverse cardiac event* - *Death* - *Myocardial infarction* - *Percutaneous coronary intervention* - *Coronary artery bypass graft* |  |  |
| **Number of outcomes in relation to the number of candidate predictors (events per variable)**   - *Major adverse cardiac event* - *Death* - *Myocardial infarction* - *Percutaneous coronary intervention* - *Coronary artery bypass graft* |  |  |
| **Notes:** | | |
| **Missing data** | **Description** *(as in report)* | **Location** *(page/figure/table)* |
| **Number of participants with any missing value**   - *History* - *Electrocardiogram* - *Age* - *Risk factors* - *Troponin* - *Outcome* |  |  |
| **Handling of missing data**  *(e.g., complete case analysis, imputation, other methods)* |  |  |
| **Notes:** | | |
| **Model performance** | **Description** *(as in report)* | **Location** *(page/figure/table)* |
| **Method used for testing model performance**  *(e.g., external validation)*   - *Temporal difference (yes/no/unclear)* - *Geographical difference (yes/no/unclear)* - *Different setting (yes/no/unclear)* - *Different investigators (yes/no/unclear)* |  |  |
| **Outcome measures**  *(e.g., risk, relative risk, absolute risk difference, sensitivity, specificity, predictive values)* |  |  |
| **A priori cut points used**  *(e.g., HEART 0-3, HEART 0-2)*  *(yes/no/unclear)* |  |  |
| **Calibration**  *(e.g., calibration plot, calibration slope, Hosmer-Lemeshow test)*  *(yes/no/unclear)* |  |  |
| **Discrimination**  *(e.g., C-statistic, D-statistic, long-rank)*  *(yes/no/unclear)* |  |  |
| **Notes** | | |
| **Results** | **Description** *(as in report)* | **Location** *(page/figure/table)* |
| **Number of outcomes for HEART score 0–3**   - *Major adverse cardiac event* - *Death* - *Myocardial infarction* - *Percutaneous coronary intervention* - *Coronary artery bypass graft* |  |  |
| **Number of outcomes for HEART score 4–10**   - *Major adverse cardiac event* - *Death* - *Myocardial infarction* - *Percutaneous coronary intervention* - *Coronary artery bypass graft* |  |  |
| **Notes:**  *(any alternate presentation of prediction model performance e.g., results for different HEART score ranges, decision analysis curve)* | | |
| **Interpretation and Discussion** | | |
| **Notes:**  *(e.g., comparison with other studies, discussion of generalisability, strengths and limitations)* | | |
